# Immunohistochemical Analysis of Rat Renal Tumours Caused by Ochratoxin A

**DOI:** 10.3390/toxins9120384

**Published:** 2017-11-28

**Authors:** Diana Herman, Peter Mantle

**Affiliations:** 1 Pathology Department, County Hospital Timisoara, Timisoara 300736, Romania; diaherman@yahoo.com; 2 Centre for Environmental Policy, Imperial College London, London SW7 2AZ, UK

**Keywords:** renal cell carcinoma, ochratoxin A, immunohistochemistry, urothelial cancer, leiomyosarcoma, rhabdomyosarcoma, Balkan endemic nephropathy

## Abstract

Experimental renal cancer caused by ochratoxin A (OTA) in rats was first defined in the US National Toxicology Program (1989) and raised questions about any aetiological role in human urinary tract tumours. A review of histopathology in several rat kidney tumours from dietary OTA in recently described London studies, augmented by clinical immunohistochemistry for the first time for this mycotoxin, establishes their renal tubular cell origin. It had been assumed that the toxin might cause the human urothelial tumours associated with Balkan endemic nephropathy, but the present study could not support this. Comparison with a similar review of a metastasising renal tumour from a female rat of the NTP study consistently shows the kidney as the primary carcinogenic site for OTA. Morphological heterogeneity of these kidney tumours as epithelioid and/or sarcomatoid is revealed. Leiomyosarcoma was also diagnosed, and rhabdomyosarcoma differentiation was observed in the exceptionally aggressive NTP female tumour. The present pilot study involving immunohistochemistry indicates need for wider review of archived tumours for experimental evidence before formulating any epidemiological basis from a rat model for OTA’s relevance to idiopathic human renal cell carcinoma. Although the NTP study concluded that females are less sensitive to OTA than males, some female tumours still had heterogeneous morphology.

## 1. Introduction

Ochratoxin A (OTA) was first characterised in South Africa [1] but found application as a significant mycotoxin in epidemics of nephropathy in the Danish bacon industry, first noted by Larsen [2] and seasonally prevalent in the 1960s and 1970s. Renal carcinogenicity was demonstrated experimentally in male mice after protracted exposure to OTA [3]. A major study followed in the US National Toxicology Program [4,5], notably demonstrating renal carcinomas, particularly in male rats. The latter generated precautionary concern for natural OTA occurrence in some foodstuffs such as cereals, coffee and wine caused by fungal spoilage of agricultural products, and precipitated current human food safety regulations for the toxin in several parts of the world [6]. A focus on the mechanisms of carcinogenicity was made in a European Commission project in the 2000s, confirming male rat sensitivity to continuous dietary OTA during most of the first half of life [7,8].

Meanwhile, following the deductions of Krogh [9] that OTA’s causal nephropathic role in Danish porcine morbidity might point to an analogous role in the human disease Balkan endemic nephropathy (BEN), the urinary tract cancers commonly associated with the human disease were shown to be of urothelial origin [10]. Soon after, the International Agency for Research on Cancer considered OTA as ‘possibly carcinogenic to humans’ [11], although no rat or mouse renal tumours caused by OTA exposure had been shown experimentally to mimic human urothelial neoplasms, whether at urothelial sites or as renal cell carcinomas in renal parenchyma. However, rat kidney tumours may be either of renal cell or urothelial cell origin, but gross distortions of many experimental OTA kidneys preclude morphological diagnosis of their origin. Some experimental kidney tumours caused by OTA are accompanied by neoplasms in other organs (e.g., lung). These have always been assumed to be metastatic from the kidney [4], but have never been subjected to contemporary clinical immunohistochemical scrutiny.

The present pilot study seeks for the first time to contribute to enhanced pathological definition of archived rat tumours, in response to chronic dietary exposure to OTA in the London studies [12,13], also through an application of clinical immunohistochemistry. Focus has been on large carcinomas from males caused by daily OTA intake in the range 30–300 µg/kg body weight, dosages much greater than in natural human exposure. Additionally, a prominent carcinoma among females of the NTP study [4] has been included for immunohistochemical comparison. Histopathology in further sections of the other nine NTP female renal tumour cases, to complement general gender-response features already illustrated [14], is also critically reviewed.

## 2. Results

### 2.1. London Males and an NTP Exceptional Female (General Features)

#### 2.1.1. Morphology on H&E Stain

The six malignant kidney tumours studied immunohistochemically (Table 1) have various morphological features, ranging from small, nodular, well-demarcated and epithelioid, to large, sarcomatoid, infiltrative and highly aggressive ones. These two morphological forms occur within the same tumour mass, either admixed or forming separate areas.

**Figure 1:**
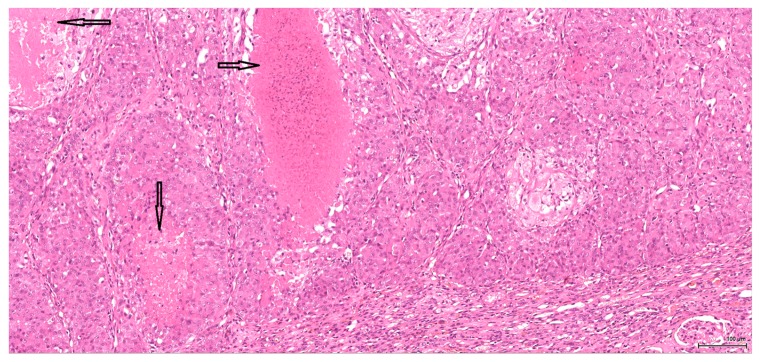
H&E: Male (case 1): epithelioid morphology, very focal clear cytoplasm, comedo-like tumour necrosis (arrows), pushing-type invasion front.

The epithelioid component combines predominantly high-grade cytology with vesicular, nucleolated, mitotically active nuclei and abundant eosinophilic cytoplasm, which only occasionally appears clear. The tumour cells are arranged in a solid pattern, with nested, alveolar and tubular architecture; rarely noted are pseudopapillary structures. Sometimes, cystic or hyaline degeneration is seen. A common finding is necrosis; tumour lobules with central necrosis (comedo-like) are frequently present. The tumour invasion front is mostly of the pushing type (Figure 1).

**Figure 2:**
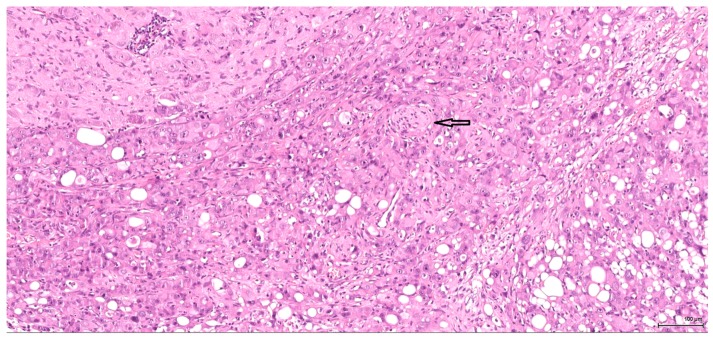
H&E: Male (case 4): high-grade tumour with aggressive behaviour, infiltrating the perirenal fat (bottom right), nerves (arrow) and part of an autonomic ganglion (top left).

A sarcomatoid pattern is encountered in five cases (2–6) in various proportions, showing spindle cell morphology and exhibiting rhabdoid features in one case. The tumour invasion front is infiltrative; entrapped glomeruli and unaffected tubuli within the tumour mass are noted. The three cases with large sarcomatoid areas (4–6, one of which had associated rhabdoid features), have an aggressive biological behaviour; vascular and neural invasion (Figure 2) are seen. These tumours are no longer organ-confined; they manifest a marked locally invasive capacity with distant metastases (lung, liver).

**Figure 3:**
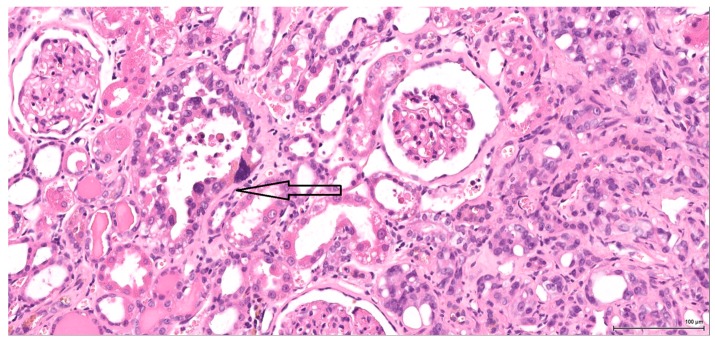
H&E: Male (case 3): marked intraepithelial cytological atypia within an enlarged tubule (arrow), adjacent to invasive tumour (right).

Two particularly interesting features have been noted, both of which independently imply a renal tubular cell origin of the tumour. In one tumour case, the infiltrative angulated tubuli and the desmoplastic reaction are suggestive of collecting-duct morphology. The second feature is found in another tumour case: a few well-defined and better-differentiated foci, revealed by immunohistochemistry, are present within the spindle cell pattern, supporting the renal cell origin of the tumour. Also supportive of a tubular cell origin is the fact that the adjacent renal parenchyma shows tubular intraepithelial dysplastic change in four cases, ranging from very focal and subtle to marked intraepithelial cytological atypia (Figure 3).

#### 2.1.2. Tumour Immunohistochemistry

**Figure 4:**
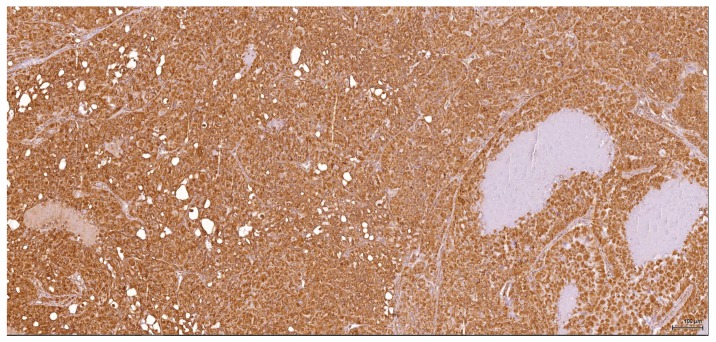
Vimentin: Male (case 2): intense and diffuse reaction.

**Figure 5:**
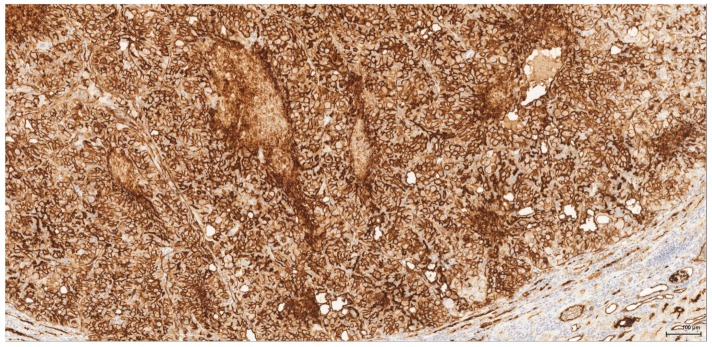
CD10: Male (case 2): intense and diffuse reaction.

**Figure 6:**
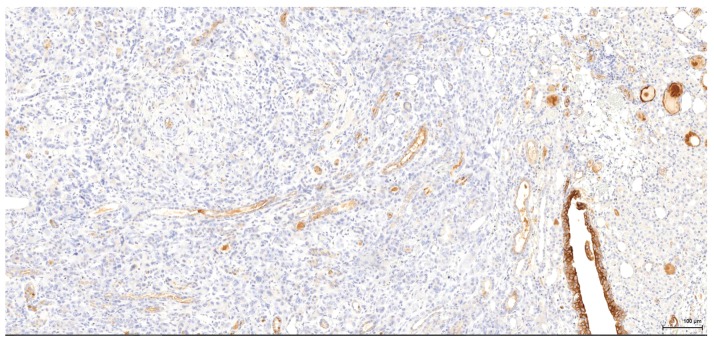
CK7: Male (case 3): tumour cells are negative, whereas residual urothelium is positive.

Immunohistochemical profiles (Table 1) point towards a renal cell origin in some cases, thus excluding a urothelial nature for these tumours. All six tumours are vimentin-positive (Figure 4); in three cases (1–3) the epithelioid component is coexpressing CD10 (Figure 5) and is negative for CK7 (Figure 6), CK20, p63 and CKHMW. This profile strongly favours renal cell origin.

The fourth case is showing an extensive sarcomatoid pattern, lacking CD10 expression and showing vimentin and pancytokeratin MNF116 expression, with a weak focal CK7 reaction, mostly with luminal pattern. This vimentin-pancytokeratin co-expression could be suggestive of a sarcomatoid carcinoma; however, the dysplastic changes found nearby indicate a renal cell origin of the tumour (renal cell carcinoma with sarcomatoid differentiation).

**Figure 7:**
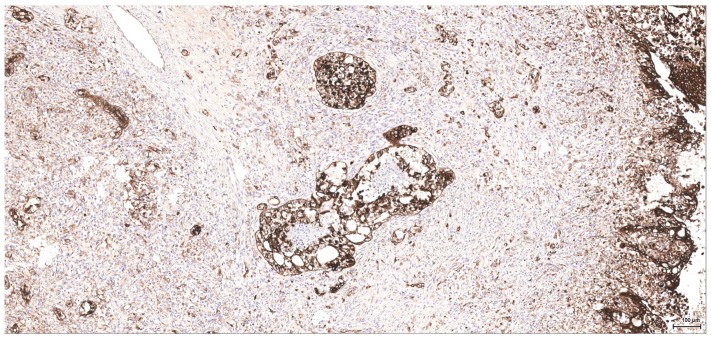
CK MNF116: Female NTP (case 5): high-grade tumour, a few better differentiated foci with minimal tubular formation.

**Figure 8:**
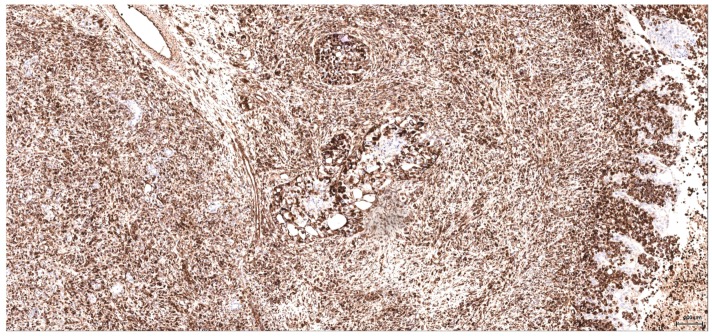
Vimentin: Female NTP (case 5): intense and diffuse reaction, including the better-differentiated foci; negative residual urothelium infiltrated by tumour (right).

**Figure 9:**
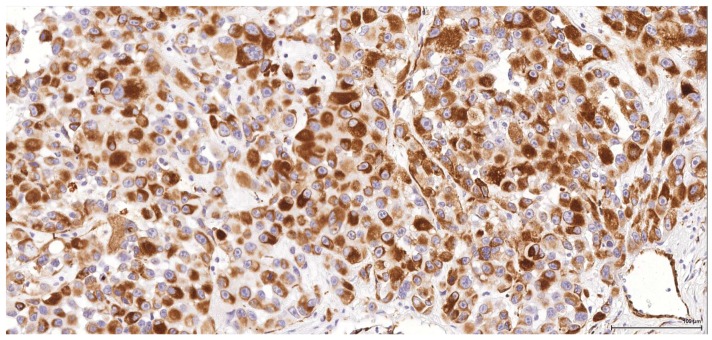
Desmin: Female NTP (case 5): rhabdoid features with eccentrically placed nuclei and intense positive reaction.

The most intriguing case (5), the only female rat in this immunohistochemical study, shows a large infiltrative tumour, involving the whole kidney, abutting and focally destroying the pelvic urothelial mucosa and invading the surrounding adipose tissue. This tumour shows high grade cytology with vesicular nucleolated nuclei, mitotically active, predominant epithelioid and spindle cell sarcomatoid morphology, including, focally, a small area with rhabdoid features, with abundant eosinophilic cytoplasm and eccentrically placed nuclei. A few well-defined foci are noted, crossing transversally the whole thickness of the kidney. These foci show a solid pattern and occasionally form tubular structures (Figure 7); immunohistochemically they express a profile similar to a conventional renal cell carcinoma (vimentin positive, as seen in Figure 8 and CD10 weakly positive, not shown). These foci might represent areas of better-differentiated tumour, supporting a renal cell origin of the tumour. However, the rhabdoid area is demonstrating intense positive reaction for vimentin and desmin (Figure 9), smooth muscle actin being negative. This area is best regarded as a heterologous differentiation along the rhabdomyosarcoma line.

**Figure 10:**
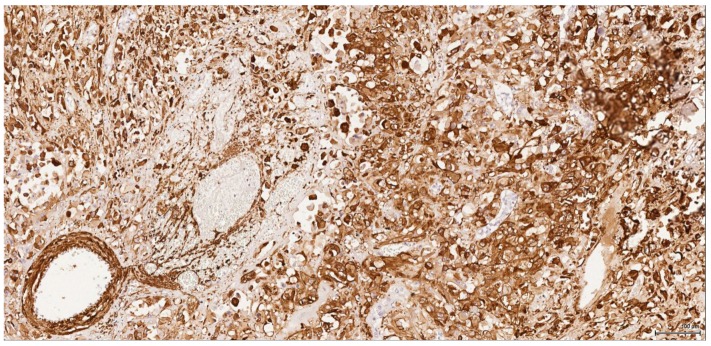
Actin: Male (case 6): intense and diffuse reaction, entrapped negative tubuli.

The last case (6) showing exclusively spindle cell morphology reveals intense and diffuse reaction for vimentin and smooth muscle actin (Figure 10), with only focal-positive expression for desmin; CKs and CD10 are negative and, therefore, based on this immune profile, this tumour is diagnosed as leiomyosarcoma. Associated tumour nodules in the lung were metastatic from the kidney, as proven immunohistochemically.

### 2.2. NTP Females

#### 2.2.1. NTP Females (Five Cases with No Tumour According to the Material Examined)

**Figure 11:**
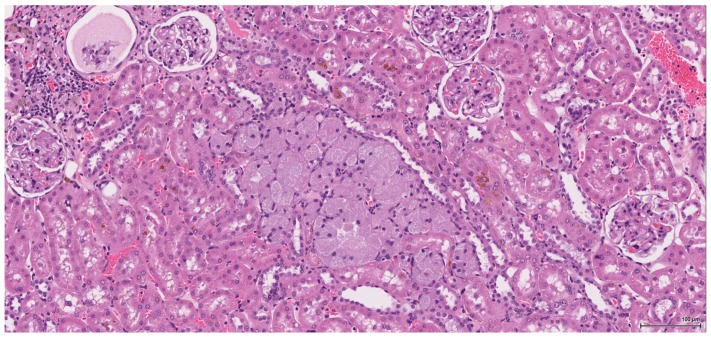
H&E: Female NTP non-tumour case: oncocytic hyperplasia (**centre**) with enlarged, finely granular, pale eosinophilic cytoplasm.

The renal lesional background is represented in most cases by different stages of chronic progressive nephropathy including degenerative and regenerative aspects: vacuolar cytoplasmic degeneration; cortical subcapsular foci of small basophilic tubuli, some of them with thickened basement membrane; compensatory enlarged hypertrophic tubuli; cystically dilated tubuli or glomeruli with glomerular metaplasia of the parietal Bowman’s capsule (typically appears in rat females after administration of androgenic compounds); casts, both hyaline and granular; variably sized cysts located within the medulla and lined by an attenuated epithelium (toxic effect or ageing related). Along with these features, regenerative tubule hyperplasia and even oncocytic hyperplasia (Figure 11) were identified in two cases. Extensive tubule necrosis is seen in one case and moderate chronic inflammatory infiltrate in another; minimal papillary urothelial hyperplasia is associated in one case.

All cases show tubuli with various degrees of cytological atypia such as markedly enlarged (karyomegaly), pleomorphic, hyperchromatic or vesicular nuclei, with prominent nucleoli. These tubuli are located at the cortico-medullary junction in the inner medulla and papilla. This type of atypia is either focal, for example, one isolated cell amongst the epithelial cells of the tubule, or sometimes the smaller cysts are lined entirely by such an atypical epithelium. However, this is not associated with proliferating activity.

#### 2.2.2. NTP Females (Five Confirmed Tumour Cases)

**Figure 12:**
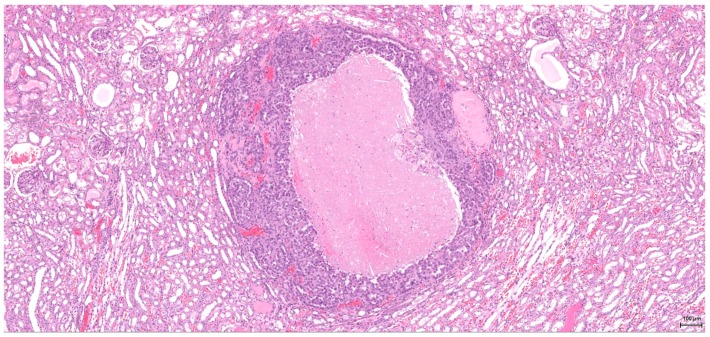
H&E: Female NTP tumour case: small, well-differentiated, low-grade tumour.

Tumour proliferation in these five cases ranges from large, poorly differentiated, metastasising tumour to small (0.5 mm) well-differentiated organ-confined lesions, which are even difficult to discriminate from atypical tubule hyperplasia. One case, carcass 1195 in [4], shows low-grade cytology and papillary architecture (Figure 12), while all others are high grade. Two cases demonstrate exclusively epithelioid morphology. Three are of mixed type (epithelioid and spindle cell), one of which has predominant sarcomatoid component. The latter is exceptional and is described in more detail above, and within Table 1 (case 5).

Tumour necrosis is seen in all but one case. Atypical tubule hyperplasia in one case is intimately associated with tumour mass, while in another case the lesion is away from the tumour. Variable degrees of focal-isolated karyomegaly are frequently seen, with morphology and distribution similar to that described in non-tumour kidney cases.

## 3. Discussion

Historically, the renal cell origin has been difficult to establish and only the present IHC could prove it. It is important to emphasize that these tumours are of renal tubular cell origin and not urothelial, as proven by immunohistochemistry and suggested by the renal intraepithelial dysplastic lesion. Morphologically the renal cell nature is not immediately obvious, the cytoplasm being rather eosinophilic and only focally clear, as expected from a conventional clear cell renal cell carcinoma. Also, the nuclei are showing prominent nucleoli, visible on low power, indicating a high nuclear grade (Fuhrman). Likewise, the frequent association of a spindle cell component (sometimes massive or even exclusive) potentially hinders the renal cell origin assessment. However, immunohistochemically, the co-expression of vimentin and CD10 and the lack of reaction for CK7, CKHMW and p63 are supportive of this origin. In normal kidney, CD10 is strongly expressed by the cytoplasm and membrane of podocytes and proximal tubular cell brush borders; in renal cell carcinoma CD10 is usually positive in clear cell (including eosinophilic, granular cell variant) and papillary type renal cell carcinomas (RCC). In addition to this unequivocal immunohistochemical profile, an indirect criterion for non-urothelial cell origin is the fact that the sarcomatoid component association is rather uncommon for urothelial neoplasms; very few cases have been described in the literature [15]. Sarcomatoid RCC is not very frequent either, but is still found in a significant percentage of RCC cases.

There is overall high grade cytology in both epithelioid and sarcomatoid areas, with high nuclear grade and necrosis; all these findings place these tumours in a poorly differentiated category. No classic conventional clear cell RCC picture has been identified; the epithelioid cells were mostly of eosinophilic granular cell type. When large amounts of spindle cell morphology are admixed, even merely associated or predominate, the degree of dedifferentiation is mirrored by the altered immune expression of CD10, which is lost. Instead, pan-CK expression occurs, which is variably co-expressed with vimentin, thus favouring the sarcomatoid nature of the tumour.

RCC with sarcomatoid features is not currently recognized as an entity as a specific type of renal parenchymal carcinoma by the most recent 2016 WHO classification of renal tumours, mainly because sarcomatoid areas can be found in all histologic subtypes of RCC [16]. Collecting duct carcinoma exhibits the highest percentage of cases with sarcomatoid change ranging from 25% to 29% in separate series, whilst clear cell RCC displays sarcomatoid change in 5.2% to 8% of cases, compared with 2% to 9% by chromophobe RCC, and 1.9% to 5.4% by papillary RCC [17].

Sarcomatoid RCC may arise out of a background of any histologic type of RCC as a manifestation of a final common dedifferentiation pathway. A pure sarcomatoid pattern is uncommon and, as recommended by the same previously mentioned guidelines, should be categorized as unclassified RCC (grade 4 unclassified carcinomas with a sarcomatoid component). The overwhelming sarcomatoid component found in these six tumours is striking because of its frequency and extent. OTA dose-factor difference does not seem to be crucial since, for example, within the same lot one tumour is organ-limited, small, nodular, well-demarcated, epithelioid and without spindle cell morphology, whilst the other one is a highly aggressive, metastasising tumour with sarcomatoid features.

Rhabdoid differentiation in RCC refers to the development of neoplastic cells that morphologically resemble rhabdomyoblasts but differ in ultrastructural features and immunophenotype (being desmin negative). The similar immunophenotype of rhabdoid and non-rhabdoid foci supports the origin of the rhabdoid cells from renal cells. Areas of rhabdoid morphology do not represent metaplastic muscle differentiation [16]. Bearing this in mind, the only case with focal rhabdoid morphology on a sarcomatoid background which does express desmin (but no SMA) might be interpreted as a heterologous differentiation along the rhabdomyosarcoma line arising in an otherwise unclassified RCC with sarcomatoid component.

Completely different in the present study is case 6, showing a spindle cell proliferation which is positive diffusely for SMA and focally for desmin. This indicates a pure mesenchymal origin, suggestive of leiomyosarcoma.

These poorly differentiated tumours have a highly aggressive course; they are usually very infiltrative, invading the surrounding local structures and metastasising at distant sites (liver, lung, mediastinum). The metastases have similar tumour morphology and are confirmed by the same immune profile. Consistently, all tumours at sites distant from a tumorous kidney were shown to have IHC profiles corresponding to the primary kidney neoplasm, confirming their metastatic status. This demonstrates the near-exclusive relationship between OTA and kidney concerning carcinogenicity in the rat, even after many months of exposure to the toxin.

Dysplasia of tubular epithelium is probably a biologic precursor of at least some RCC, and it is also a strong support for the renal cell origin. In this study it was noticed within the adjacent renal parenchyma in four cases, ranging from mild to severe intraepithelial cytological atypia, similar to carcinoma in situ; interestingly, this lesion has been found occasionally in the contralateral kidney as well.

Premalignant precursor lesions of RCC are not well characterized, but intratubular epithelial dysplasia defined morphologically by crowded, enlarged and pleomorphic nuclei has been described by other authors [18]. However, this finding remains understudied and outside the current recommendations for the pathological reporting of RCC. It warrants further study that will provide new insights into the pathogenesis, biologic behaviour, and natural history of RCC. In the present context it is worth mentioning that even the most subtle morphological changes (easily missed on H&E stain) could be potentially spotted by ribosomal p-S6 protein immunohistochemical expression, as demonstrated by one of our previous studies [19]. Karyomegaly is presumed to result from nucleic acid replication without nuclear division. It is characterised by marked enlargement of nuclei observed predominantly in the proximal convoluted tubule cells and has been associated with chemical administration [20]. Karyomegaly per se is considered a preneoplastic lesion and should be graded and reported. In our study karyomegaly, regarded as a dysplastic intraepithelial lesion, is a widespread change found in all cases, both male and female, and can be graded from mild to severe. The localisation is similar to that of the preneoplastic lesion below; sometimes one can follow a preneoplastic pathway, so to speak, along one collecting duct from the main tumour through the entire papilla.

Atypical tubule hyperplasia is a tubule-confined epithelial proliferation of more than two to three cell layers, with variable degrees of cytoplasmic and nuclear pleiomorphism. It may occur spontaneously, in association with chronic progressive nephropathy, or as a result of chemical administration. Atypical tubule hyperplasia usually arises from the proximal convoluted tubule, but a distal or collecting duct origin is also possible. It is considered a putative preneoplastic lesion that is part of the continuum, leading to neoplasia [20]. In our study, this proliferative (intra)tubular precursor lesion is seen either at the periphery of the tumour mass or as a minute lesion (0.1 mm) at a distant site.

Although only half of the elderly females in which renal tumour was recorded in the NTP study [4] showed similar evidence in the sections provided for the present study, there is no cause to modify tumour incidence data. The small size of most neoplasms will readily affect ease of recognition in different section planes. Nevertheless, the present review confirms the marked rat gender difference in sensitivity to OTA as a renal carcinogen, and raises questions about the factor(s) affecting the limited proliferation of tumours in a female’s kidney during two years of gavage delivery of OTA. Nevertheless, occasional microscopic lesions in renal parenchyma, identified as ‘atypical tubule hyperplasia’, but not distorting the kidney, were probably not clinically significant for those animals.

Concerning the gender differential in the OTA dose-response, the perception of a mechanism involving OTA binding to male-specific rat (and presumably mouse) serum alpha 2u-globulins and augmented delivery to nephron epithelia via glomerular filtration, as in alpha 2u-nephropathy, has been described [14]. It could make only the female rat dose-response renal carcinogenicity data potentially applicable to humans, since humans do not have these globulins. Unfortunately, exclusive temporary access to a commercial OTA primary antibody for that study has precluded subsequent confirmation.

The relevance of some experimental whole rat data to humans may be uncertain as a model, but for OTA the rat has been the best available. Studies on putative molecular tumorigenic mechanisms have in recent years used the sophisticated methodologies that have become available. However, no clear relevant picture has emerged to explain how tumour occurrence is mainly unilateral with highly focal origin in just a few of the thousands of separate nephrons, and that requires the insults from the ingestion of the mycotoxin well into the µg/kg b.w. range during most of the first year of life. OTA has been administered experimentally in vitro to tissue-cultured cells or kidney homogenates without knowledge of the relevant kidney epithelial cell exposure to OTA during in vivo tumorigenesis, because that is unknown. Previously, an analysis of several London OTA/rat carcinomas did not identify evidence of mutations in the rat orthologues of human familial renal cell carcinoma genes VHL and FLCN (Ricketts and Maher, personal communication), and genome-wide genetic sequencing studies are currently in progress. From the present immunohistochemical review of tumour heterogeneity a direct model for humans is uncertain, especially since a better affinity with spontaneous renal tumours arising in the Eker rat has recently been recognised also immunohistochemically [20]. A comprehensive IHC study of all archived OTA/rat tumours from London dietary exposure experiments now seems desirable to quantify the spectrum of histopathologies within and across their experimental occurrence. This might be augmented from the NTP Archives, comparing its oral gavage delivery of OTA with the dietary protocol used in London. However, there remains the mysterious aetiology of complex histopathological findings in the bizarre NTP female rat studied here, set in the context of the markedly lower sensitivity of female rats and mice to OTA renal carcinogenicity. We also recommend preparing male mouse renal tumours, based on [3], for full genetic analysis of an animal with a defined genome and using less of the expensive OTA.

Therefore, a provisional conclusion of the present pilot study is that the rat fails to provide any model support for an aetiological role for OTA in urothelial cancers sometimes associated with Balkan endemic nephropathy. Further, a predictive animal-model role for the rat in human RCC aetiology seems at present insecure. Notably in the particular case of the late Palle Krogh, whose premature demise from a metastasising RCC might have come from professional experimental exposure to OTA for several years, the primary renal tumour was diploid [21] in marked histopathological contrast with the aneuploidy of OTA/rat carcinomas [22]. This makes an aetiological connection between experimental rat and natural human difficult for OTA, particularly since experiment needs a vastly greater dosage of the mycotoxin than is naturally available.

## 4. Materials and Methods

### 4.1. Male Rat Renal Cancer Cases for IHC

Archived paraffin-embedded tumorous kidneys from Fischer males (Table 1; cases 1–4) and some other tissues from another recently published study [12], were selected partly to reflect the wide morphological range of prominent renal tumours caused by chronic exposure to OTA (300 µg/kg b.w., twice the high dose for females above). Selection also allowed comparison between continuous dietary OTA exposure for 85 weeks (cases 3 and 4) and freedom from such exposure for a year after only 42 weeks of exposure in the first year of life (cases 1 and 2). In the latter cases, most or all of tumour development would have been during absence of circulating OTA. Case 6 is the most prominent amongst the few renal tumours described for Fischer males [11] following continuous dietary OTA exposure (50 µg/kg b.w.; similar to that for case 5 above), and is considered together with metastases that are also from archived paraffin-embedded tissues. Renal tumours in cases 2, 3 and 6 had already been designated deoxyribonucleic acid (DNA) aneuploid [21] (in which, specifically for case 6 here, see Table 1, rat 5, Figure 1L and Figure 2E). The extensive renal distortion/destruction by most of these tumours has precluded exclusion of their origin from transitional cell epithelia of the renal pelvis until the present study.

### 4.2. NTP Female Rat Tumours from OTA

The 1989 NTP study [4] reports unilateral renal neoplasms in eight out of 50 Fischer rats given the high OTA diet by gavage (mean daily dose 150 µg/kg b.w.). All were recorded at the end of the two-year study, in the absence of overt morbidities; six were clinically designated as adenomas and two as carcinomas. In another part of the NTP two-year study, renal neoplasms were seen in two rats given one-third of the high dose, and were designated either as adenoma or as carcinoma. The latter was exceptional in rats for all chronic OTA exposure studies to date in being revealed only after 43 weeks of OTA exposure and accompanied by neoplasms at multiple sites, interpreted in the 1980s as metastases from kidney. For assistance in some gender studies in London [13], H&E sections of 10 cases had been provided by NTP Archives in 2006 and were therefore available for review here. Additionally, glass slide-mounted paraffin sections of an array of the putative metastases in the 43 weeks case were provided in 2016 (Table 1, case 5) and used for immunohistochemistry.

### 4.3. Immunohistochemistry

Where necessary, 3-µm sections were cut from paraffin blocks. Sections on charged slides were studied immunohistochemically, using both automatic and manual procedures in three different locations: Peterborough City Hospital, Peterborough, UK; St George’s Hospital, London, UK, and; County Hospital, Timisoara, Romania.

The following antibodies were used: CK AE1/AE3, clone AE1/AE3 (Dako); CK MNF 116, clone MNF 116 (Dako); CK HMW, clone 34 βE12 (Dako, Novocastra); CK7, clone OV-TL 12/30 (Dako); CK20, clone Ks 20.8 (Dako); p63, clone DAK-p63 (Dako); Vim, clone V9 (Dako, Novocastra); CD10, clone 56C6 (Dako); Desmin, clone D33 (Dako); Smooth Muscle Actin, clone 1A4 (Dako); TTF-1, clone SPT24 (Novocastra), and; S100, polyclonal (Dako, Novocastra). The antibody titer varies depending on the commercial form used: fixed/prediluted (the so-called ready-to-use variant), respectively, the optimal dilution of the concentrated form, individually adjusted according to the internal protocol of each laboratory. For S100, CD10, Vimentin and some cytokeratins (CKs), the immune reactions were repeated in all three locations by different methods with the same results. The rationale of using the above listed markers is explained in the discussion chapter under the renal cell origin section. Some of these antibodies have been applied selectively on a few cases only, where differential diagnosis was considered (e.g., TTF-1, Desmin and SMA, see Table 1). From the multitude of markers used in this study, only those including a confident internal control within the section (see Figure 6) have been listed; the others, for example, HMB45 or Melan A (both negative in case 6), have been discharged from the list.

The staining protocol varied from site to site, but the main steps were:

1. Endogenous enzyme block with Peroxidase Blocking Reagent for 5 min.
2. Primary antibody incubated for 30 min or overnight.
3. Secondary antibody/reagent link for 15 min.
4. Labelled polymer/HRP for 20 min.
5. Substrate-chromogen (DAB) for 10 min.
6. Counterstain with Haematoxylin for 5 min.

The brown immune reaction product of DAB chromogen is cytoplasmic and/or membranar for most of the listed antibodies, nuclear for p63 and TTF-1, and cytoplasmic and nuclear for S100. The immunoreactivity was evaluated by a percentage of positive tumour cells as diffuse for ≥50% and focal for <50%; the intensity of reaction was appreciated as weak or intense compared to control.

## Figures and Tables

**Table 1:** Summary of rat kidney tumour context, morphology, biological behaviour and immunohistochemistry findings.

Cases	OTA Weeks	OTA Dose * µg/kg b.wt.	Age Weeks	Morphology	Dysplastic Change	Metastases	Vim	CD 10	MMF 116	Desmin	Others
1 UK Male	42	300 (feed)	107	Epithelioid	No	No	+	+	−	−	
2 UK Male	42	300 (feed)	105	Epithelioid & limited spindle cell	Minimal	No	+	+	−	−	
3 UK Male	85	300 (feed)	93	Epithelioid & spindle cell/sarcomatoid	Yes	No	+	+	−	−	
4 UK Male	85	300 (feed)	93	Epithelioid & sarcomatoid	Yes, bilateral	Yes	+	−	+	−	CK7 var TTF1 − Actin −
5 NTP Female	43	50 by gavage	52	Epithelioid & sarcomatoid focally rhabdoid	No	Yes	+	− (focally weak)	variable	+ areas	Actin − S100 −
6 UK Male	83	50 (feed)	91	Sarcomatoid	Yes, only contra-lateral	Yes	+	−	−	+ focally	Actin +

* Daily dose for UK cases; mean daily dose for the NTP case. Ochratoxin A (OTA); United Kingdom (UK).
